# Extraction cellulose from corn-stalk taking advantage of pretreatment technology with immobilized enzyme

**DOI:** 10.1039/d1ra07513f

**Published:** 2022-01-05

**Authors:** Chunhua Lou, Yongli Zhou, An Yan, Yang Liu

**Affiliations:** College of Materials Science and Engineering, Qiqihar University Qiqihar 161006 China chunhualou@163.com; Heilongjiang Provincial Key Laboratory of Polymeric Composition Materials 161006 China

## Abstract

In this paper, the corn-stalk cellulose (CSC) was extracted from the corn-stalk pretreated by the immobilized enzyme which was prepared using xylanase and laccase. The immobilization rate was strongly affected by such conditions as the carrier concentration and the carbodiimide (EDC) concentration. The structure of CSC was characterized by Fourier infrared (FTIR). X-ray diffraction (XRD) was used to verify the crystallinity of CSC. The crystallinity of CSC was 74.13%, which was increased by 25.95% than that of the corn-stalk. The morphologies of the corn-stalk pretreated by the immobilized enzyme and the CSC extracted were confirmed by scanning electron microscopy (SEM). The experimental results showed that the cellulose content of the cellulose obtained only by acid–base method was 85.46%, and that of the cellulose obtained by immobilized enzyme was 96.72%. What indicated that the immobilized enzyme pretreatment could loosen the cell well of corn-stalk and partly remove lignin and hemicelluloses from corn-stalk, which was beneficial for the further extraction of cellulose by other treatment methods.

## Abstract 2

In this paper, the corn-stalk cellulose (CSC) was extracted from the corn-stalk pretreated by the immobilized enzyme which was prepared using xylanase and laccase.

## Graphical abstract

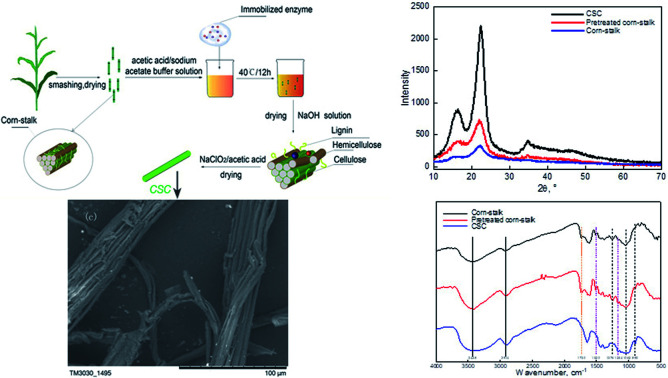

## 1 Introduction

Corn is one of the most important crops in the world. In addition to fruits, corn had whiskers, husks, stalks and other by-products, of which the annual output of stalks was as high as about 350 million tons in China. In our country, most farmers chose to burn corn-stalk, which would not only waste resource but also pollute the air environment. Therefore, effective utilization of corn-stalk has become a hot research topic at present.^1^ Cellulose extraction should be undoubtedly one of the effective ways to develop and utilize the corn-stalk.

Cellulose, a kind of biomass resource, is a sort of renewable natural organic polymer with the most abundant in natural world.^2^ The cellulose is an annular macromolecular polysaccharide formed by the d-pyran glucose group, which molecular formula is (C_6_H_10_O_5_)_*n*_. Its molecular weight is so large that its degree of crystallinity is high.^3^ Cellulose has the characteristics of porous and large specific surface area. In addition, its surface contains lots of hydroxyl groups, which is very convenient for modifying. The corresponding cellulose derivatives can be obtained by physical, chemical, biological and other methods, which have a very high development potential and commercial utilization value. With the development of human society, the demand for cellulose as a green and renewable resource will increase day by day, and the utilization of cellulose will continue to become the focus of scientific research.^4^

Cellulose inside plants, the main component of plant cell walls, can't be used directly because it is surrounded by hemicelluloses and lignin, closely bound together by covalent bonds, hydrogen bonds and van der Waals forces, which make it difficult to separate.^5^ Cellulose can be extracted effectively only when the three substances are effectively separated.

The extraction methods of corn-stalk cellulose mainly include physical methods and chemical methods. In practical application, two or more methods are mostly used in combination to give play to each other's advantages to improve the effect of cellulose separation and extraction. Physical methods, mainly including mechanical crushing, steam explosion,^6–8^ high-energy radiation^9,10^ and ultrasonic^11–14^ auxiliary extraction methods, *etc.*, are generally used in cellulose pretreatment process or auxiliary process. Their purpose is to remove lignin, hemicelluloses and other components that have protective actions on cellulose. However, the physical methods require optimization of treatment time and strength to prevent the breaking of the cellulose chain. Chemical methods are the process of using chemicals to break the link between lignin and cellulose and dissolve hemicelluloses at the same time. Chemical methods include acid–base method,^15–17^ organic solvent method,^18–22^ ionic liquid method^23–30^ and so on. Acid–base method is the commonly used method to extract corn-stalk cellulose. Its advantages include simple extraction process, high extraction efficiency, good thermal stability, good crystallinity, easy control of reaction conditions and low cost. Using acid–base method to extract corn-stalk cellulose is beneficial to achieve large-scale industrial production and have broad development space.

In this paper, the immobilized enzymes prepared using xylanase and laccase were used to pretreat stalk for the first time, and then the acid–base method was used to extract cellulose, which greatly improved the effect of cellulose separation and extraction.

## 2 Experimental

### 2.1 Preparation of immobilized enzyme

In this study, Eudragit resin, Eudragit L 100 supplied by Yingchuang Special Chemical Co. Ltd., China, was selected for carrier. A certain amount of Eudragit resin and distilled water were added in the beaker, then, stirred enough to obtain carrier turbid liquid. The 4 mol L^−1^ NaOH solution was added dropwise with stirring until the pH value of this carrier turbid liquid got to around nine. At this time, due to Eudragit L 100 was completely dissolved, the carrier turbid liquid turned to transparent carrier solution. The 10% acetic acid aqueous solution was added dropwise in this solution with stirring till the pH value decreased to about six. A certain amount of 1-ethyl-3-dimethyl amino propyl carbodiimide hydrochloride (EDC) was added and stirred to a uniform mixture, in which the 10% acetic acid aqueous solution was dropped until the pH value decreased to 4.8, then, 0.1 mol L^−1^ acetic acid/sodium acetate buffer solution was added. The beaker was stored at 4 °C for 12 hours after sealed. Next, xylanase and laccase were added when the carrier solution regain to room temperature. The 10% acetic acid aqueous solution was added dropwise in this mixture solution with stirring till the pH value decreased to four or so after stored at 4 °C for 24 hours. At this moment, the gel-like sediment which was the immobilized enzyme after being centrifuged was present in the beaker.

**Fig. 1:**
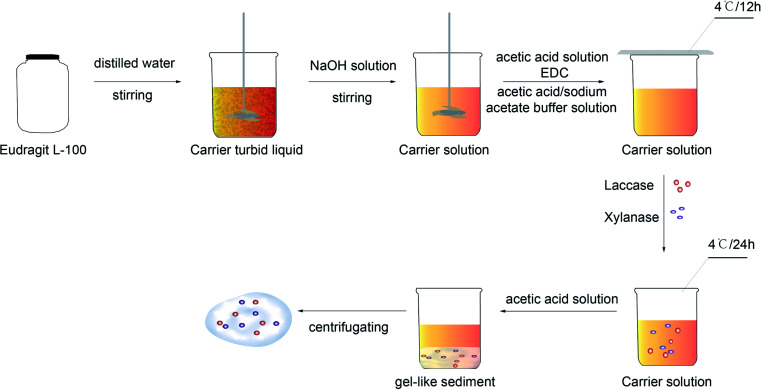
Preparation process chart of immobilized enzyme.

The preparation process of immobilized enzyme is shown as Fig. 1.

### 2.2 Pretreatment corn-stalk with immobilized enzyme

**Fig. 2:**
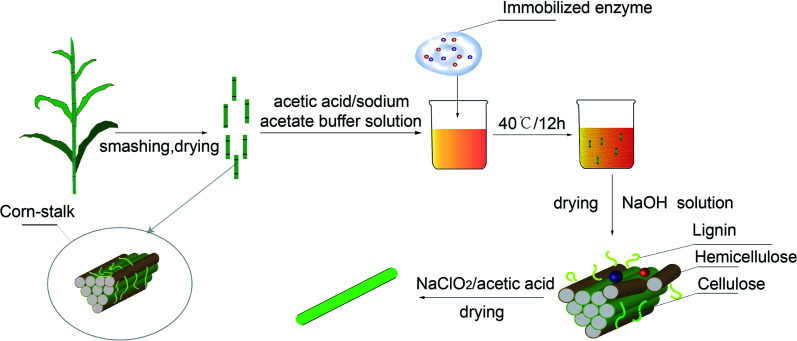
Process flow chart of stalk pretreatment and cellulose extraction by immobilized enzyme.

Firstly, corn-stalk which was washed and got rid of silt with distilled water was smashed to powder with nearly 180 μm after being dried. Immobilized enzyme and corn-stalk powder were added in a beaker in which 0.1 mol L^−1^ acetic acid/sodium acetate buffer solution was added in. The pretreatment corn-stalk was obtained after suction filtrating and drying the mixture which was stored at 40 °C for 12 hours. The process flow of stalk pretreatment is shown as Fig. 2.

### 2.3 Extraction cellulose

The pretreated corn-stalk was added in the NaOH solution and then filtrated and dried after being stirred at 60 °C for 2 hours. The corn-stalk cellulose was obtained during the production treated by base above was treated by the mixed solution of sodium chlorite and acetic acid at 70 °C for 1 hour. The process flow of cellulose extraction by immobilized enzyme is shown as Fig. 2.

### 2.4 Test of the xylanase activity

According to GB/T 23874-2009, the amount of enzyme required for degradation of 1 μmol reducing sugar from 5 mg mL^−1^ xylan solution per minute defines one unit of xylanase activity (*μ*) at the condition of PH is 5.5 and temperature is 37 °C.

The specific method of xylanase activity test is as follows:

5.0 mL DNS reagent was added in the 4.0 mL acetic acid–sodium acetate buffer solution, which was heated at a boiling water bath for 5 minutes. Next, the mixed solution was added water to 25 mL after cooling to the room temperature in a tap-water bath, which was the standard blank sample.

2.0 mL xylose standard solution of different concentration was suck up into a graduated test tube, respectively. Next, 2.0 mL buffer solution and 5.0 mL DNS reagent were added in each test tube, respectively. Then, the test tube was heated at the boiling water bath for 5 minutes after electromagnetic oscillating for 3 to 5 seconds. The mixed solution was added water to 25 mL after cooling to the room temperature in the tap-water bath. The absorbance *A* was measured at 540 nm with the standard blank sample as blank control.

Then the standard curve of xylose is *y* = 1.172*x* − 0.0002, *R*_2_ = 0.9998. Where *y* is the concentration of xylose; *x* is the absorbance.

2.0 mL diluted enzyme solution and 5 mL DNS reagent were orderly added in a graduated test tube and electromagnetic oscillated for 3 to 5 seconds, then, kept constant temperature at 37 °C for 30 minutes and heated at the boiling water bath for 5 minutes after 2.0 mL xylan solution was added in. Next, the mixed solution was added water to 25 mL after cooling to the room temperature in the tap-water bath. The absorbance *A*_B_ was measured at 540 nm with the standard blank sample as blank control.

2 mL diluted enzyme solution and 2 mL xylan solution were orderly added in a graduated test tube and electromagnetic oscillated for 3 to 5 seconds, which was kept constant temperature at 37 °C for 30 minutes. 5 mL DNS reagent was decanted in the test tube to terminate the enzymatic hydrolysis. Then, the test tube was heated at the boiling water bath for 5 minutes. Next, the mixed solution was added water to 25 mL after cooling to the room temperature in the tap-water bath. The absorbance *A*_E_ was measured at 540 nm with the standard blank sample as blank control.

So, the computation formula of enzyme activity is shown as formula (1):1
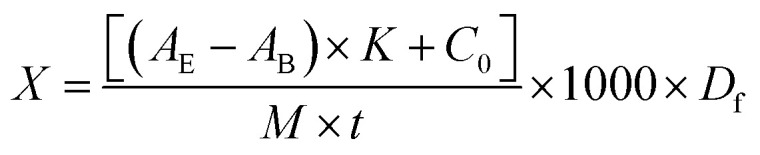
where *X* is the xylanase activity (μ g^−1^); *A*_2_ is the absorbance of enzyme solution; *A*_1_ is the absorbance of enzyme blank sample; *K* is the slope of the standard curve; *C*_0_ is the intercept of the standard curve; *M* is the molecular wright of xylose (150.2); *t* is the reaction time of enzymatic hydrolysis (min); 1000 is the transforming factor; *D*_f_ is the dilution ratio of the samples.

### 2.5 Test of the laccase activity

One unit of the laccase activity is defined that the laccase breaks down 1 μmol 2,2′-azine-di(3-3-ethylbenzothiazole-6-sulfoacid) (ABTS) per minute, which makes the free radical concentration of ABTS increase from *a* to *b*.

The specific method of the laccase activity test is as following:

0.1 mL diluted enzyme solution was added in 2.9 mL ABTS solution. The absorbance time from one point (*A*_1_) to another point (*A*_2_) was recorded, and then the laccase activity was calculated according to the definition of other enzyme activity.

Firstly, the curve of relationship of ABTS free radical and absorbance is *y* = 1.793*x* − 0.0002, *R*_2_ = 0.9998. Where *y* is the concentration of ABTS free radical; *x* is the absorbance.

So, the computation formula of enzyme activity is shown as formula (2):2
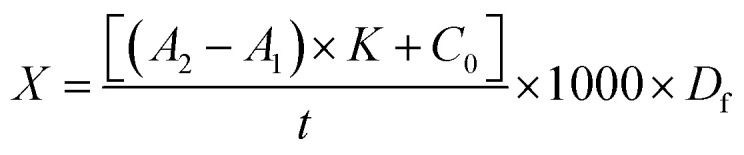
where *X* is the laccase activity (μ g^−1^); *A*_1_ and *A*_2_ are two end values of the absorbances of enzyme solution; *K* is the slope of the standard curve; *C*_0_ is the intercept of the standard curve; *t* is the time when the absorbance value changes from *A*_1_ to *A*_2_ (min); 1000 is the transforming factor; *D*_f_ is the dilution ratio of the samples.

### 2.6 Measurement of cellulose content

The dried sample about 1 g (*m*_0_) was accurately weigh and put it into a 250 mL clean and dry flask, in which 25 mL nitrate–ethanol mixture was added. Then, the reflux condensing tube was loaded on the flask and heated in boiling water bath for 1 hour, subsequently, the solvent was removed by vacuum filtration. If the fiber did not turn white, the above steps were repeated. The residue was washed repeatedly with water until the residue was neutral. Finally, the residue was washed twice with anhydrous ethanol and vacuum filtrated completely. The residue was placed in an oven at 105 °C until its mass was constant (*m*_1_), and then in a muffle furnace at 600 °C until its mass was constant (*m*_2_).

The computational formula of cellulose content is shown as formula (3):3
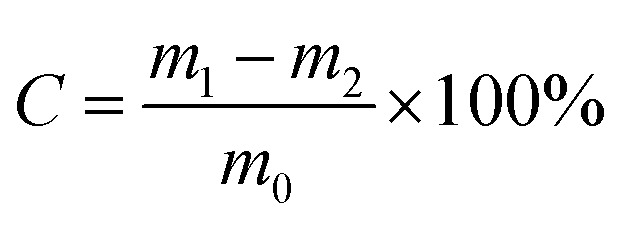
where *C* is the cellulose content.

### 2.7 Characterization

FTIR spectra of samples were recorded on a FTIR in a range of wave numbers from 4000 to 500 cm^−1^, using a Frontier fourier transform infrared (PE Instruments).

X-ray diffraction (XRD) analyses was performed at room temperature using a D8 Bruker in a range of diffraction angles from 5 to 80° at the scanning speed of 2° min^−1^.

The surface morphology of corn-stalk analysis was undertaken by TM3030 type Scanning Electron Microscope (Hitachi, Japan).

The vitality tests of xylanase and laccase were performed using Lamda 35 spectrometer (PE Instruments).

## 3 Results and discussion

### 3.1 Experiments on preparation of immobilized enzyme

The immobilization rate would be a criterion with which to judge the quality of immobilized enzyme. Higher this rate is, better the catalytic effect of immobilization enzyme is. The calculation formula of immobilization rate is shown as formula (4):4
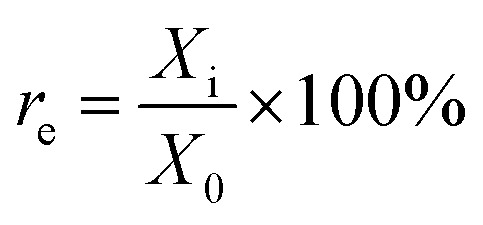
where *r*_e_ is the immobilization rate; *X*_i_ is the activity of the immobilization; *X*_0_ is the initial activity of the enzyme.

#### 3.1.1 The effect of carrier concentration on enzyme immobilization rate

**Fig. 3:**
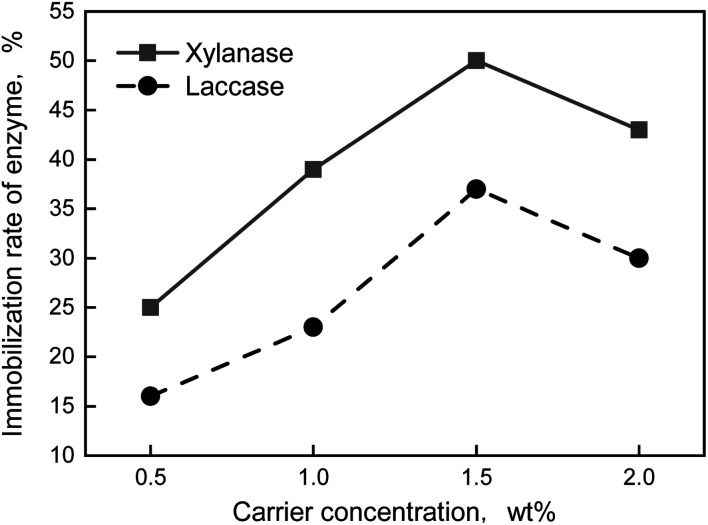
Effect curves of carrier concentration on immobilization rate of enzyme.

The carrier concentration has a great influence on enzyme immobilization rate. The immobilization rate would reduce owing to the carrier cannot carry enzyme when the carrier concentration was too low. While the active center of enzyme would be coved by excess carrier which would influence the enzyme activity when the carrier concentration was high. From Fig. 3, the enzyme immobilization rate increase at first and then decrease with increasing the carrier concentration. And the immobilization rates of both xylanase and laccase were all the highest when the carrier concentration was 1.5 wt%.

#### 3.1.2 The effect of EDC concentration on enzyme immobilization rate

**Fig. 4:**
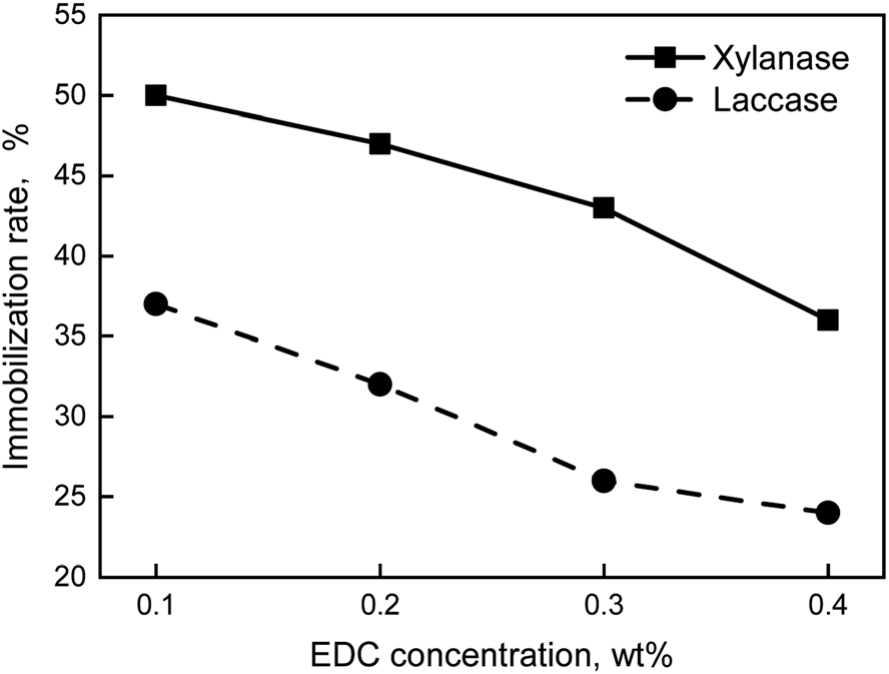
Effect curves of EDC concentration on enzyme immobilization rate.

EDC, a kind of water-soluble carbodiimide, could activate carboxyl and promote to synthesis amide when the pH value was in the range of 4.0 to 6.0. Adding EDC could make the carrier deposit easier, at the same time, also make zymoprotein molecule combine with the carrier more advantageously. Seen from Fig. 4, the immobilization rates of both xylanase and laccase were all decrease with increasing the DEC concentration. The reason could be that the more EDC added, the more possible the condensation would be.

In conclusion, the optimum formula of preparation of immobilization enzyme was that the carrier was 1.5 wt% and EDC was 0.1 wt%. The immobilization enzyme was prepared by the formula above, thus, the immobilization rate of xylanase and laccase was 49% and 37%, respectively.

### 3.2 Experiment on pretreatment corn-stalk with immobilized enzyme

#### 3.2.1 The effect of pH value on degradation efficiency

**Fig. 5:**
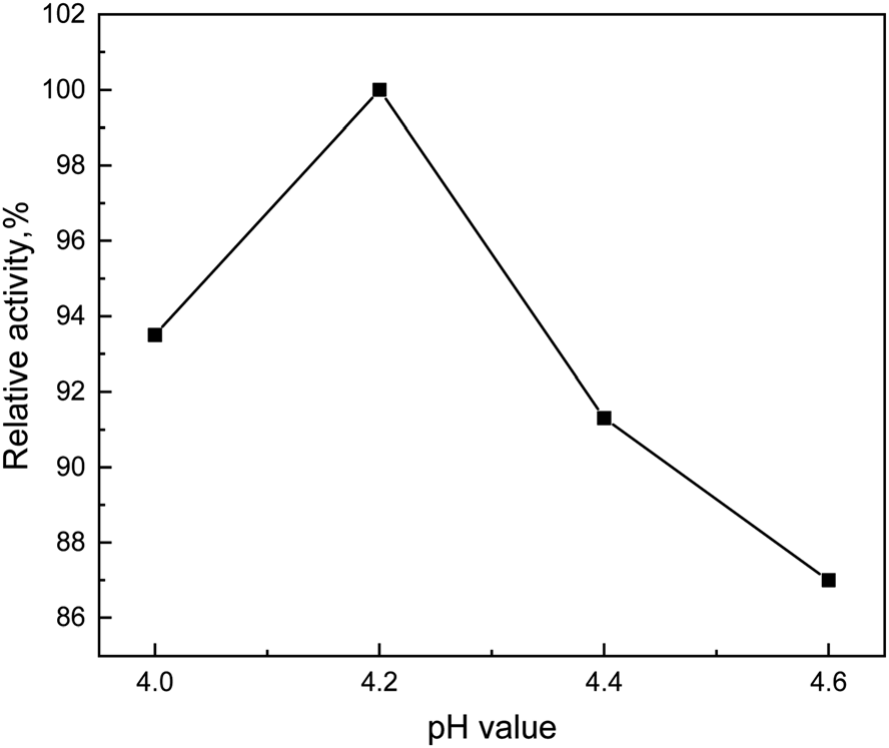
Effect curve of pH value on degradation efficiency.

The pH value could affect the degree of dissociation of both essential groups at the active center of the enzyme and the substrate, which would affect the catalytic ability of the enzyme to the reaction substrate. Therefore, there should have an appropriate range of the pH value for enzymes. Whether the pH value was greater than or less than the suitable pH value, the enzyme activity would be broken, and even made irreversible damage. Hence, the enzyme activity and the degradation efficiency of lignin would attain the maximum value at the optimal pH value. Fig. 5 showed that the relative activity of enzyme increased first and then decreased with the increase of pH value. When the pH value is 4.2, the relative activity of enzyme reached the highest, which indicated that the enzyme activity and the lignin dissolved were the best.

#### 3.2.2 The effect of temperature on degradation efficiency

**Fig. 6:**
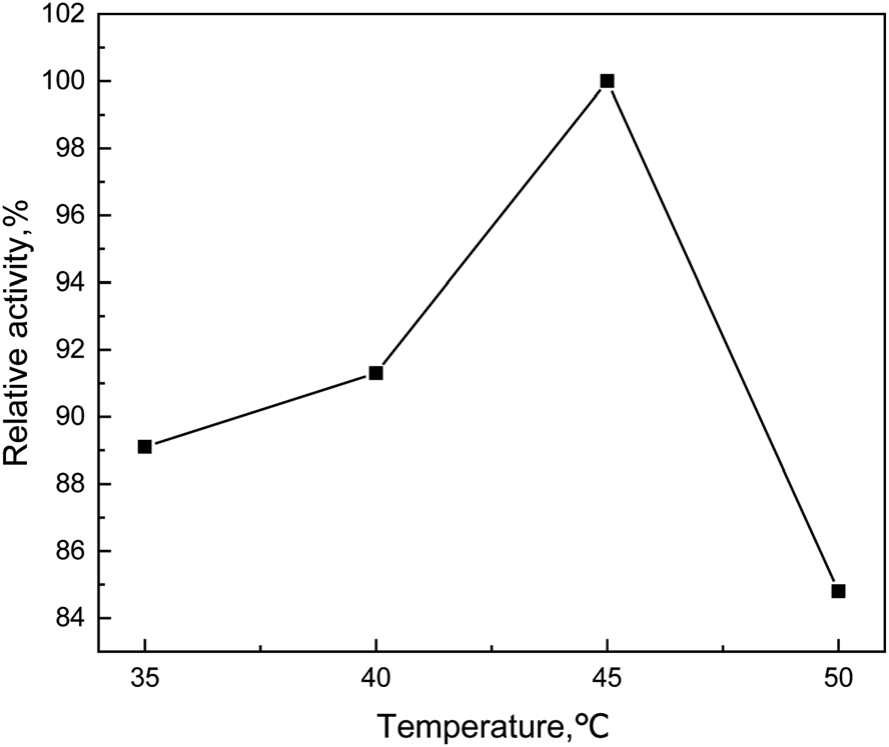
Effect curve of temperature on degradation efficiency.

Due to the percentage of activated molecules increased, both the rate of chemical reaction and the catalytic effect would be improved with the increase of temperature. However, as a kind of protein, enzyme was very sensitive to temperature. The function of enzyme could not perform fully, which would make the catalytic effect lower at low temperature. The activity of the enzyme increased with the increase of temperature, nevertheless, the enzyme would inactivate because it denatured once the temperature exceeded the appropriate temperature. As shown in Fig. 6, the relative activity of enzyme increased first and then decreased with the increase of temperature. When the temperature is 45 °C, the relative activity of enzyme reaches the maximum point, which proved that the enzyme activity and the lignin dissolved were the best.

#### 3.2.3 The effect of the concentration of the corn-stalk on degradation efficiency

**Fig. 7:**
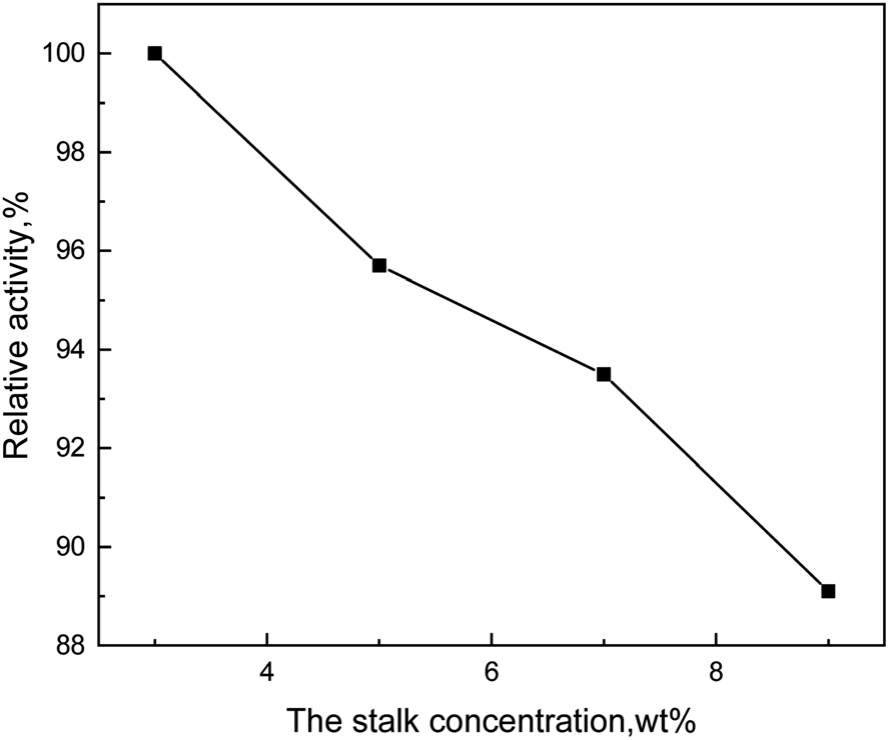
Effect curve of stalk concentration on degradation efficiency.

Immobilized enzymes, fixed on the carrier, are different from free enzymes which are not limited by the carrier and can swim around in the reaction system and fully contact with the reaction substrate. Hence, immobilized enzymes have no way to move freely so that the contact opportunity and the contact area between the enzymes and the corn-stalk are restricted. In addition, the uneven stirring would decrease the contact area between the immobilized enzyme and the corn-stalk, which would reduce the catalytic effect. Fig. 7 showed that the relative activity of enzyme gradually declined with the increase of stalk concentration, which demonstrated that the catalytic effect of immobilized enzyme so effective that more lignin was dissolved.

#### 3.2.4 The effect of time on degradation efficiency

**Fig. 8:**
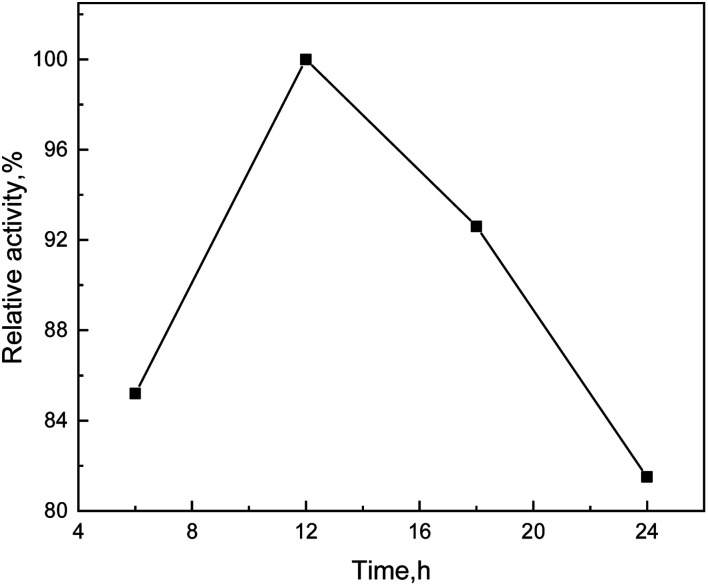
Effect curve of time on degradation efficiency.

Reaction time is an important factor of enzyme pretreatment. That is, a short time is insufficient for reaction, while a long time will waste resources and affect production efficiency. From Fig. 8, with the increase of reaction time, the relative activity of enzyme had a trend of increasing first and then decreasing. The relative activity of enzyme reached the maximum when the reaction being continued for 12 hours, which indicated that the lignin dissolved was the most. When the reaction time was more than 12 hours, the relative activity of enzyme decreased. This was probably because the content of lignin in the reaction system was so high that it was sucked back to the corn-stalk, which resulted in the content of lignin in the enzymolysis solution declined.

### 3.3 The measurement of cellulose content

As could be seen from Table 1, the cellulose content obtained only by acid–base treatment with un-pretreatment was 85.46%, while that obtained by immobilized enzyme pretreatment was 96.72%. The results manifested that the immobilized enzymes could destroy the cell wall of corn-stalk, the protective layer of biomass on the surface of corn-stalk, which effectively loosened the corn-stalk. Thus, the acid and base solution could more easily enter the inside of the corn-stalk, which made the extraction reaction more sufficient. Therefore, the purity of cellulose was higher than that obtained only by acid and base method with un-pretreatment.

**Table 1:** Cellulose content by different approach

	Acid–alkali method	
	Un-pretreated	Pretreated with immobilization enzyme
Cellulose content	85.46%	96.72%

### 3.4 The characterization analysis of CSC

#### 3.4.1 FT-IR analysis

**Fig. 9:**
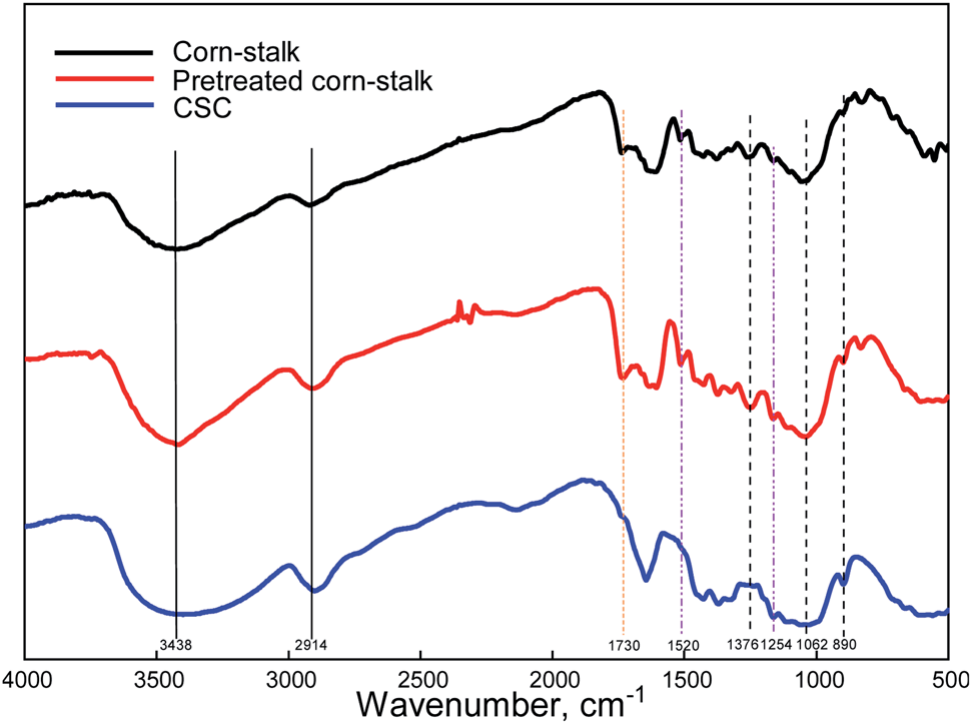
Infrared spectra of corn-stalk, pretreated corn-stalk and CSC.

Corn-stalk contains lignin, hemicelluloses and cellulose, all of which contain a lot of hydroxyl and methylene, and the corresponding characteristic peaks appear at 3438 cm^−1^ and 2914 cm^−1^, respectively. The characteristic peaks of lignin are 1520 cm^−1^ and 1254 cm^−1^, which are benzene ring and carbonyl group in lignin, respectively. The characteristic peak of hemicelluloses is 1730 cm^−1^, which is a non-conjugated carbonyl group on the acetyl group. While the characteristic peaks of cellulose are at 1376 cm^−1^, 1062 cm^−1^ and 890 cm^−1^, which are C–H and C–O in cellulose, respectively, and –C–O–C on pyran ring and glycoside bond, respectively. From Fig. 9, pretreated corn-stalk also had all characteristic peaks because most of the lignin and hemicelluloses remained. The characteristic peaks of lignin and hemicelluloses have disappeared, indicating that both lignin and hemicelluloses have been completely removed by pretreatment and acid–base treatment. So, high purity cellulose was obtained.

#### 3.4.2 X-ray diffraction analysis

**Fig. 10:**
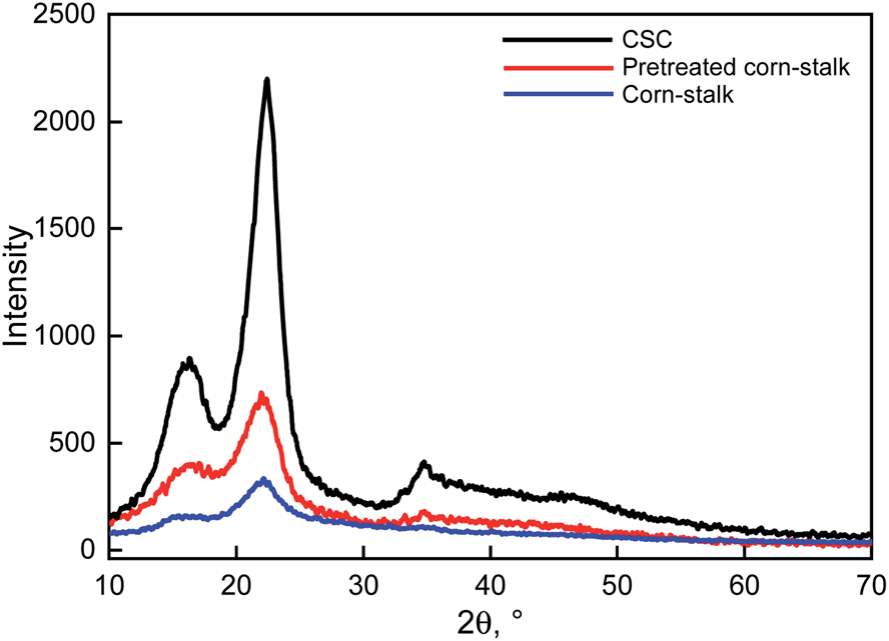
XRD patterns of corn-stalk, pretreated corn-stalk and CSC.

From Fig. 10, the diffraction peak positions of the three specimens were basically the same. The peak of 15.9° corresponds to the 101 crystal plane, the peak of 22.3° corresponds to the 002 crystal plane, and the peak of 34.4° corresponds to the 040 crystal plane, which are typical structures of cellulose I. The results showed that the corn-stalk did not change the crystal structure of cellulose after pretreatment and acid–base treatment. The peaks of corn-stalk at 15.9°, 22.3° and 34.4° are not obvious, because corn-stalk contains a lot of lignin and hemicelluloses, which would affect the expression of peak. After the corn-stalk pretreated by immobilization enzyme, the three peaks slightly increased, which was because the pretreatment might partly get rid of lignin and hemicelluloses. The lignin and hemicelluloses were basically completely removed from corn-stalk after pretreatment and acid–base treatment, as a result that the diffraction peaks of CSC were the narrowest and sharpest.

The crystallinity of corn-stalk, corn-stalk pretreated and CSC was 48.18%, 53.78% and 74.13%, respectively. Since lignin and hemicelluloses are amorphous, the improvement of crystallinity also indicated that lignin and hemicelluloses have been removed from corn-stalk. That is, pretreatment by immobilization enzyme should destroy the surface of corn-stalk and degrade part of lignin and hemicelluloses. Only if during these two methods combined to treat the corn-stalk successively, the lignin and hemicelluloses could be completely removed from corn-stalk.

#### 3.4.3 SEM analysis

**Fig. 11:**
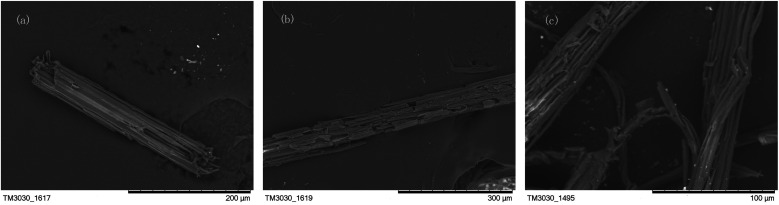
SEM images of corn-stalk (a), pretreated corn-stalk (b) and CSC (c).

Fig. 11(a) was the morphology diagram of corn-stalk. It could be observed that normal corn-stalk was covered by a complete biomass shell composed of lignin, hemicelluloses, pectin, impurities, *etc.* Its structure was integrity and its surface was smooth.

Fig. 11(b) was the morphology diagram of the pre-treated corn-stalk by the immobilization enzyme. It could be observed that the shell structure outside the corn-stalk was no longer smooth with many breakages, even many holes were appeared. This was because xylanase could degrade the xylan in hemicelluloses, thereby destroy the hemicelluloses. At the same time, laccase could break the chemical bonds in lignin and degrade it. Therefore, in the process of pretreatment, hemicelluloses and lignin in the stalk cell wall were degraded by xylanase and laccase, respectively, which resulted in the stalk cell wall destructed.

Fig. 11(c) showed the morphology of corn-stalk cellulose extracted by ourselves. From Fig. 11(c), the filamentous structure of the cellulose of the corn-stalk could be clearly observed. The probably reason was after immobilized enzyme pretreatment and acid–base treatment, hemicelluloses, lignin, pectin and other substances in corn-stalk were basically completely removed. Under the combined action of acid and alkali, the biomass husk outside the corn-stalk had been destroyed completely, and the fiber structure inside had also been completely exposed.

## 4 Conclusions

The immobilization enzyme was successfully prepared using Eudragit L 100 as carrier, xylanase and laccase as enzyme preparation and EDC as crosslinking agent. Then corn-stalk was pretreated with this immobilization enzyme. The optimum conditions of immobilized enzyme pretreatment were obtained by single factor experiment, that is, the reaction was conducted at 45 °C for 12 hours when the pH value was 4.2 and the stalk concentration was 3%. And the cellulose content obtained by immobilized enzyme pretreatment was 96.72%.

## Conflicts of interest

There are no conflicts to declare.

## Supplementary Material
